# Incidence and risk factors of metabolic syndrome among Royal Thai Army personnel

**DOI:** 10.1038/s41598-022-19024-8

**Published:** 2022-09-20

**Authors:** Boonsub Sakboonyarat, Ram Rangsin, Murray A. Mittleman

**Affiliations:** 1 Department of Military and Community Medicine, Phramongkutklao College of Medicine, Bangkok, Thailand; 2 Department of Epidemiology, Harvard T.H. Chan School of Public Health, Boston, Massachusetts USA

**Keywords:** Risk factors, Epidemiology, Public health

## Abstract

Metabolic Syndrome is a clustering of obesity, hyperglycemia/insulin resistance, dyslipidemia, and hypertension. We aimed to determine the incidence of metabolic syndrome among Royal Thai Army (RTA) personnel and its risk factors. We conducted a retrospective cohort study using data from 2017 to 2021. Metabolic syndrome was defined by NCEP ATP III (2005 Revision). A total of 98,264 participants were enrolled in the present study. The overall incidence rate of metabolic syndrome was 3.7 per 100 person-year (95% CI 3.7–3.8). The statistically significant risk factors for metabolic syndrome included male sex (aHR 1.40; 95% CI 1.29–1.51), age > 35 years, current alcohol consumption, and no exercise. When stratified by sex, the incidence rate of metabolic syndrome among participants aged ≥ 45 years was higher than those aged < 35 years with aHR 6.34; 95% CI 6.01–6.70 for males and aHR 9.59; 95% CI 7.55–12.19 for females. Our data demonstrated that metabolic syndrome is a common health issue, especially among RTA personnel over 35 years. Alcohol consumption and sedentary behavior played an essential role in facilitating metabolic syndrome in this study population and are potential targets for intervention to enhance primary prevention of the sequelae of metabolic syndrome.

## Introduction

Metabolic syndrome is a clustering of obesity, hyperglycemia/insulin resistance, dyslipidemia (DLP), and hypertension (HT)^1, 2^. It identifies patients at high risk of developing type 2 diabetes (T2D) and atherosclerotic cardiovascular diseases (ASCVD)^2, 3^. Furthermore, metabolic syndrome is associated with a 2.4 times higher mortality rate compared with those without metabolic syndrome^3^.

The prevalence of metabolic syndrome in each geographic region is diverse. In the Asia–Pacific region, a recent study reported that the prevalence of metabolic syndrome ranged from 11.9 to 37.1%^4^ while its prevalence in South Asia was 26.1%^5^. Several studies reported different incidences of metabolic syndrome accounting for 3.1, 21.0, and 22.9 per 1000 person-years in Spain^6^, Japan^7^ and Taiwan^8^, respectively. In Thailand, recent studies have reported that the prevalence of metabolic syndrome among Thai males is 11.7 to 25.8%, while among females, prevalence is 8.2 to 26.3%^9–13^.

Military personnel constitute a specific population group exhibiting different behavior risk factors from the civilian population. Moreover, the institutional structures of the military convey harmful behaviors among personnel and weaken their desire to embrace healthier habits^14^. In Thailand, a recent study reported that the prevalence of current alcohol consumption among military personnel was approximately 70%^15^ higher than that among Thai civilians (68.5%)^16^. In addition, related evidence among Royal Thai Army (RTA) personnel emphasized that obesity prevalence has continuously risen from 2017 to 2021^15^. A related meta-analysis reported that the overall estimated prevalence of metabolic syndrome among military personnel was 8.0%, according to the National Cholesterol Education Programme Adult Treatment Panel III (NCEP-ATP III) criteria^17^. However, the evidence on the incidence of metabolic syndrome and risk factors among the military population, including in Thailand, was scarce. Further evidence is needed concerning behavioral risk factors to enhance primary prevention of metabolic syndrome and its sequelae, including noncommunicable diseases (NCDs) and ASCVD.

Nationwide, approximately 130,000 Royal Thai Army (RTA) personnel participate in a yearly health examination provided by the RTA Medical Department^18^. In the present study, we investigated the incidence of metabolic syndrome among RTA personnel using the physical health examination in the RTA personnel database from 2017 to 2021. Importantly, sex is now evolving as a significant factor in developing metabolic dysregulation^19^. Furthermore, effect modification among risk factors influencing metabolic syndrome development may hold public health significance^20, 21^. Therefore, we also evaluated sex- and age-specific associations between behavioral factors and the incidence of metabolic syndrome among RTA personnel.

## Methods

### Study design and participants

**Figure 1:**
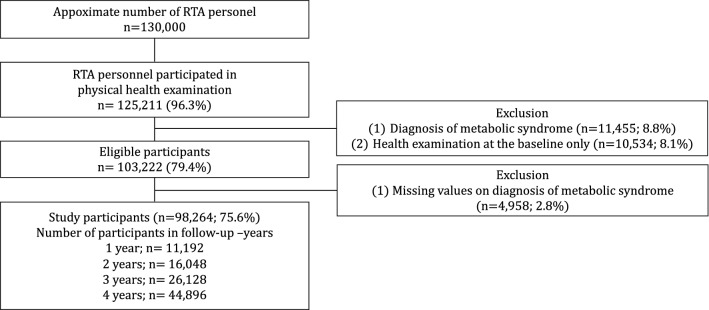
Flow chart of the study**.**

We conducted a retrospective cohort study using data from 2017 to 2021. We retrieved data from the annual health examination database of RTA personnel after obtaining approval from the RTA Medical Department in Bangkok, Thailand. The missions of the RTA Medical Department include providing healthcare services, promoting health, and preventing disease for both military personnel and Thai civilians. The RTA Medical Department provides annual health examinations for RTA personnel through the Army Institute of Pathology, Armed Forces Research Institute of Medical Sciences and 37 RTA hospitals nationwide. The records are then reported to the RTA Medical Department in Bangkok. Eligible participants consisted of RTA personnel aged 18 to 60 years nationwide who had health examination records from 2017 to 2020. Of the approximately 130,000 RTA personnel, 125,211 (96.3%) participated in physical health examinations. Participants with a history of metabolic syndrome (n = 11,455; 8.8%) and those undergoing physical health examination at baseline only (n = 10,534; 8.1%) were excluded. A final target of 103,222 (79.4%) participants without a diagnosis of metabolic syndrome participated in the baseline review. We excluded 4958 (2.9%) participants because they had missing data concerning the diagnosis of metabolic syndrome. Therefore, a total of 98,264 (75.6%) participants without a diagnosis of metabolic syndrome at baseline were enrolled in the present study and followed up until 2021. The flow of the study is presented in Fig. 1.

### Data collection

Annually, the Army Institute of Pathology, Armed Forces Research Institute of Medical Sciences and 37 RTA hospitals provide health examinations for RTA personnel. A self-report guide was created using a standardized case report form to obtain demographic characteristics, comorbidities and behavioral risk factors, including age, sex, health scheme, smoking status, history of alcohol consumption and exercise. The comorbidities included a history of HT, DLP and T2D.

The region of residence consisted of Bangkok, the north, northeast, central and south. The health scheme of the participants included Civil Servant Medical Benefits, Social Security and Universal Coverage. Behavioral risk factors were obtained from the self-reported questionnaire. Smoking status was divided in four groups including (1) never smoked, (2) ex-smoker defined as being smoke-free for 12 months, (3) irregular current smoker and (4) regular current smoker. Alcohol consumption was categorized in four groups including (1) never consumed, (2) exdrinker defined as alcohol-free for 12 months, (3) irregular current drinker and (4) regular current drinker. A history of T2D, HT and DLP comorbidity of study participants was defined using the information from the responses to the questionnaire, (1) “Have you ever been diagnosed with T2D or taken antihyperglycemic drugs?”, (2) “Have you ever been diagnosed with HT or taken antihypertensive drugs?” and (3) “Have you ever been diagnosed with DLP or taken lipid-lowering drugs?”.

The annual health examination dataset at baseline and follow-up also included anthropometric measurements of waist circumference, weight, height, systolic blood pressure and diastolic blood pressure. The information on laboratory testing and anthropometric measurements came from several RTA hospitals nationwide providing the measurements, which trained technicians performed, and the standard and quality of services of all RTA hospitals were certified by Healthcare Accreditation Institute, Thailand. Waist circumference was measured at midline level (between the inferior margin of the ribs and the superior border of the iliac crest), with a plastic tape^22^. The participant’s body weight was measured in kilograms and the height in centimeters. Blood pressure (BP) was measured using an automatic blood pressure monitor by an operator trained in standardized technique following the Thai guidelines on HT treatment. The participants were advised to avoid caffeine and smoking for at least 30 min before measuring. Two measurements were taken, and the average was recorded^23^. Laboratory data included fasting plasma glucose, triglycerides, high-density lipoprotein cholesterol.

We defined metabolic syndrome according to NCEP ATP III (2005 Revision)^24^, requiring at least three of the following components: (1) abdominal obesity (waist ≥ 102 cm (≥ 40 inches) for men ≥ 88 cm (≥ 35 inches) for women), (2) triglycerides ≥ 150 mg/dL or pharmacologic treatment, (3) HDL cholesterol ≤ 40 mg/dL for men or 50 mg/dL for women or pharmacologic treatment, (4) systolic blood pressure ≥ 130 mmHg or diastolic blood pressure ≥ 85 mmHg or taking antihypertensive medication, and (5) fasting plasma glucose ≥ 100 mg/dL or pharmacologic treatment.

### Statistical analysis

We performed statistical analyses using Stata, Version 17 (StataCorp. 2021, College Station, TX) and (2) SPSS, Version 27.0 (IBM Corp. 2020. Armonk, NY, USA). We calculated the frequency distribution of baseline characteristics, behavioral data and comorbidities to describe the study sample. Due to the nature of an observational study, the information on some variables was missing, including smoking status (5.0%), alcohol consumption (4.5%) and exercise (5.7%). However, the study consisted of a large sample size; therefore, the existing data would be included in the analysis. Categorical variables including sex, age groups, regions, health scheme, smoking status, history of alcohol consumption and exercise history were presented as percentages. We calculated the person-time of observation for each participant as the duration between the participant's baseline data and the year at which metabolic syndrome occurred or the end of the study (2021), whichever occurred first. We calculated incidence rates of metabolic syndrome with 95% confidence intervals (CI) per 100 person-years of observation, and used the log-rank test to compare the incidence rates of metabolic syndrome across characteristics. Cox proportional hazard regression analysis was used to investigate behavioral and demographic risk factors for metabolic syndrome and presented the magnitude of associations as unadjusted and adjusted hazard ratios (HR) with 95% confidence intervals. A two-sided *p*-value less than 0.05 was considered statistically significant. We also conducted a sensitivity analysis to assess the incidence rates of metabolic syndrome according to the International Diabetes Federation (IDF) 2005 and explored the association between incidence rates of metabolic syndrome and behavioral factors.

### Ethics considerations

The study was reviewed and approved by the Institutional Review Board, RTA Medical Department, Bangkok, Thailand in compliance with international guidelines such as the Declaration of Helsinki, the Belmont Report, CIOMS Guidelines and the International Conference on Harmonization of Technical Requirements for Registration of Pharmaceuticals for Human Use—Good Clinical Practice (ICH—GCP) (Approval number S067h/64). Because we used secondary data, the Institutional Review Board, RTA Medical Department granted a waiver of informed consent.

## Results

### Baseline characteristics

Table 1 shows the baseline demographic, behavioral, and clinical characteristics of the 98,264 RTA personnel included in the study population. The majority (91.1%) were males. The average age of the study participants was 35.6 ± 11.0 years. Approximately one-third (32.1%) of participants were current smokers. The baseline prevalence of regular alcohol use was 7.4%, while the prevalence of irregular alcohol intake was 63.3%. Regular exercise at baseline was reported by 63.3% of study participants.

**Table 1:** Baseline characteristics of RTA study participants (2017–2020).

Baseline Characteristics	*n* (%)
	or mean ± SD
**Total**	98,264
**Sex**	
Male	89,527 (91.1)
Female	8737 (8.9)
**Age (years)**	
mean ± SD	35.6 ± 11.0
< 35	55,496 (56.5)
35–44	18,577 (18.9)
≥ 45	24,191 (24.6)
**Regions**	
Bangkok	15,191 (15.5)
Central	28,962 (29.5)
Northeast	20,136 (20.5)
North	19,186 (19.5)
South	14,789 (15.1)
**Health Scheme**	
Civil Servant Medical Benefits	96,579 (98.3)
Social Security	1119 (1.1)
Universal Coverage	566 (0.6)
**Smoking status**	
Never	46,737 (50.1)
Ex-smoker	16,679 (17.9)
Current smoker (irregular)	13,080 (14.0)
Current smoker (regular)	16,866 (18.1)
**Alcohol drinking**	
Never	18,356 (19.6)
Ex-drinker	9172 (9.8)
Current drinker (irregular)	59,391 (63.3)
Current drinker (regular)	6935 (7.4)
**Exercise**	
No	6750 (7.3)
Irregular exercise	27,280 (29.4)
Regular exercise	58,625 (63.3)
**Waist circumference (cm)**	
mean ± SD	83.5 ± 9.1
**Fasting plasma glucose (mg/dL)**	
mean ± SD	98.9 ± 30.6
**Triglyceride (mg/dL)**	
mean ± SD	147.1 ± 110.1
**HDL-Cholesterol (mg/dL)**	
mean ± SD	57.4 ± 16.8
**Systolic blood pressure (mmHg)**	
mean ± SD	125.7 ± 14.9
**Diastolic blood pressure (mmHg)**	
mean ± SD	76.5 ± 11.5

### Incidence of metabolic syndrome among RTA personnel

A total of 11,178 (11.4%) RTA personnel developed metabolic syndrome during the follow-up period, representing an incidence rate of 3.7 per 100 person-years (95% CI 3.7–3.8). The incidence rates among males and females were 3.8 per 100 person-years (95% CI 3.7–3.8) and 3.2 per 100 person-years (95% CI 2.9–3.4), respectively. Table 2 shows incidence of metabolic syndrome among RTA personnel by characterirtics.

**Table 2:** The incidence of metabolic syndrome among RTA personnel by demographic and behavioral factors.

	Person-years of observation	No. of metabolic syndrome	Incidence rate / 100 person-years	95% CI	*p*-value
**Total**	301,256	11,178	3.71	3.69–3.80	
**Sex**					< 0.001
Female	27,310	859	3.15	2.94–3.36	
Male	273,946	10,319	3.77	3.69–3.84	
**Age (years)**					< 0.001
mean ± SD					
< 35	170,631	2100	1.23	1.18–1.28	
35–44	57,256	3210	5.61	5.42–5.80	
≥ 45	73,369	5868	8.00	7.80–8.21	
**Regions**					< 0.001
Bangkok	46,815	1782	3.81	3.63–3.99	
Central	86,718	3670	4.23	4.10–4.37	
Northeast	61,144	2463	4.03	3.87–4.19	
North	62,292	1893	3.04	2.91–3.18	
South	44,287	1370	3.09	2.93–3.26	
**Health Scheme**					0.004
Civil Servant Medical Benefits	296,753	11,034	3.72	3.65–3.79	
Social Security	3238	87	2.69	2.18–3.32	
Universal Coverage	1265	57	4.51	3.48–5.84	
**Smoking status**					< 0.001
Never	144,810	5671	3.92	3.82–4.02	
Ex-smoker	51,436	1838	3.57	3.41–3.74	
Current smoker (irregular)	39,304	1201	3.06	2.89–3.23	
Current smoker (regular)	50,420	1739	3.45	3.29–3.62	
**Alcohol drinking**					0.001
Never	56,638	2130	3.76	3.60–3.92	
Ex-drinker	27,424	1077	3.93	3.70–4.17	
Current drinker (irregular)	183,583	6511	3.55	3.46–3.63	
Current drinker (regular)	19,442	768	3.95	3.68–4.24	
**Exercise**					< 0.001
No	21,468	838	3.90	3.65–4.18	
Irregular exercise	83,614	3451	4.13	3.99–4.27	
Regular exercise	179,517	6136	3.42	3.33–3.50	

*95% CI* 95% confidence interval.

### Risk factors of metabolic syndrome among RTA personnel

Multivariable adjusted hazard ratios from the Cox model are shown in Table 3. After mutually adjusting for demographic, behavioral, and clinical characteristics, the incidence rate of metabolic syndrome was higher for males than females (adjusted HR 1.40; 95% CI 1.29–1.51), RTA personnel over age 35 years, those residing in Cental and Northeast, and those who reported current alcohol consumption. On the other hand, former smokers had a lower incidence rate of metabolic syndrome than lifelong non-smokers. Finally, those who reported regular exercise had a lower incidence rate of metabolic syndrome than sedentary participants (adjusted HR 0.79; 95% CI 0.74–0.86). There was a significant effect modification (*p*-value = 0.0001) between sex and age on the incidence rate of metabolic syndrome.

**Table 3:** Univariable and multivariable analysis of the association between demographic, behavioral, and clinical factors and the incidence of metabolic syndrome among RTA personnel.

Factors	Unadjusted HR	95% CI	*p*-value	Adjusted HR	95% CI	*p*-value
**Sex**						
Female						
Male	1.19	1.11–1.28	< 0.001	1.40	1.29–1.51	< 0.001
**Age (years)**						
< 35						
35–44	4.56	4.32–4.82	< 0.001	4.53	4.27–4.79	< 0.001
≥ 45	6.50	6.18–6.83	< 0.001	6.52	6.19–6.87	< 0.001
**Regions**						
Bangkok						
Central	1.11	1.05–1.18	< 0.001	1.40	1.32–1.49	< 0.001
Northeast	1.06	1.00–1.13	0.06	1.27	1.19–1.36	< 0.001
North	0.81	0.76–0.86	< 0.001	1.05	0.98–1.12	0.202
South	0.81	0.76–0.87	< 0.001	1.06	0.97–1.15	0.189
**Health Scheme**						
Civil Servant Medical Benefits						
Social Security	0.72	0.58–0.88	0.002	0.85	0.68–1.06	0.157
Universal Coverage	1.14	0.88–1.47	0.34	1.48	1.14–1.94	0.004
**Smoking status**						
Never						
Ex-smoker	0.91	0.87–0.96	0.001	0.87	0.82–0.92	< 0.001
Current smoker (irregular)	0.78	0.73–0.83	< 0.001	0.94	0.88–1.00	0.05
Current smoker (regular)	0.88	0.83–0.92	< 0.001	0.95	0.90–1.00	0.07
**Alcohol drinking**						
Never						
Ex-drinker	1.04	0.97–1.12	0.31	1.09	1.01–1.18	0.03
Current drinker (irregular)	0.94	0.90–0.99	0.02	1.08	1.02–1.14	0.01
Current drinker (regular)	1.04	0.95–1.13	0.40	1.21	1.11–1.32	< 0.001
**Exercise**						
No						
Irregular exercise	1.05	0.97–1.13	0.21	0.98	0.90–1.05	0.54
Regular exercise	0.87	0.81–0.93	< 0.001	0.79	0.74–0.86	< 0.001

*HR* Hazard ratio, *95% CI* 95% confidence interval.

With study participants stratified by sex (Table 4), the higher age, the higher the incidence rate of metabolic syndrome in both sex. In males, the incidence rate of metabolic syndrome was significantly higher among those with a current alcohol intake, while there is no significant association in females. In addition, males who had a history of regular exercise had a lower incidence rate of metabolic syndrome than sedentary participants; however, it was not significantly different among females.

**Table 4:** Multivariable analysis of the association between demographic, behavioral, and clinical factors and the incidence of metabolic syndrome among RTA personnel, stratified by sex.

Factors	Male			Female		
	Adjusted HR	95% CI	*p*-value	Adjusted HR	95% CI	*p*-value
**Age (years)**						
< 35						
35–44	4.55	4.29–4.83	< 0.001	4.80	3.72–6.20	< 0.001
≥ 45	6.34	6.01–6.70	< 0.001	9.59	7.55–12.19	< 0.001
**Regions**						
Bangkok						
Central	1.41	1.33–1.50	< 0.001	1.43	1.20–1.70	< 0.001
Northeast	1.29	1.20–1.38	< 0.001	1.19	0.97–1.46	0.09
North	1.05	0.98–1.13	0.19	1.16	0.90–1.49	0.26
South	1.04	0.96–1.14	0.34	1.64	1.19–2.25	0.002
**Health Scheme**						
Civil Servant Medical Benefits						
Social Security	1.11	0.81–1.52	0.51	0.83	0.59–1.15	0.26
Universal Coverage	1.72	1.28–2.32	< 0.001	1.05	0.59–1.87	0.88
**Smoking status**						
Never						
Ex-smoker	0.89	0.84–0.95	< 0.001	0.29	0.18–0.47	< 0.001
Current smoker (irregular)	0.94	0.88–1.00	0.06	0.90	0.43–1.92	0.79
Current smoker (regular)	0.95	0.90–1.00	0.07	1.81	0.93–3.55	0.08
**Alcohol drinking**						
Never						
Ex-drinker	1.13	1.04–1.23	0.004	1.15	0.83–1.61	0.40
Current drinker (irregular)	1.09	1.03–1.16	0.003	1.10	0.94–1.30	0.23
Current drinker (regular)	1.23	1.12–1.35	< 0.001	0.82	0.34–2.00	0.67
**Exercise**						
No						
Irregular exercise	0.97	0.89–1.05	0.41	1.06	0.79–1.40	0.71
Regular exercise	0.78	0.73–0.85	< 0.001	0.93	0.70–1.23	0.62

*HR* hazard ratio, *95% CI* 95% confidence interval.

Table 5 shows factors associated with incidence rate of metabolic syndrome stratified by age. The incidence rate of metabolic syndrome was higher in males than females (adjusted HR 1.81, 1.66, and 1.18) among participants aged $\le$ 35, 35–44, and ≥ 45 years, respectively. Regular alcohol intake was associated with the higher incidence rate of metabolic syndrome among RTA personnel aged 35–44 and ≥ 45 years but was not associated with the younger RTA personnel aged 35 years. Participants aged $\le$ 35 and ≥ 45 years who reported regular exercise had a significantly lower incidence rate of metabolic syndrome than those with a sedentary lifestyle; however, there was no association among RTA aged 35–44 years.

**Table 5:** Multivariable analysis of the association between demographic, behavioral, and clinical factors and the incidence of metabolic syndrome among RTA personnel, stratified by age.

Factors	< 35 years			35–44 years			≥ 45 years		
	Adjusted HR	95% CI	*p*-value	Adjusted HR	95% CI	*p*-value	Adjusted HR	95% CI	*p*-value
**Sex**									
Female									
Male	1.81	1.43–2.30	< 0.001	1.66	1.43–1.93	< 0.001	1.18	1.07–1.30	0.001
**Regions**									
Bangkok									
Central	1.48	1.23–1.79	< 0.001	1.61	1.44–1.81	< 0.001	1.37	1.27–1.47	< 0.001
Northeast	1.60	1.33–1.94	< 0.001	1.54	1.36–1.75	< 0.001	1.13	1.05–1.23	0.003
North	1.62	1.34–1.96	< 0.001	1.28	1.12–1.46	< 0.001	0.81	0.74–0.89	< 0.001
South	1.31	1.06–1.60	0.01	1.16	0.99–1.36	0.07	0.95	0.84–1.08	0.44
**Health Scheme**									
Civil Servant Medical Benefits									
Social Security	0.72	0.38–1.37	0.32	1.06	0.69–1.63	0.79	0.90	0.67–1.20	0.46
Universal Coverage	1.51	0.71–3.21	0.28	1.95	1.17–3.27	0.01	1.52	1.08–2.14	0.02
**Smoking status**									
Never									
Ex-smoker	1.03	0.91–1.18	0.60	0.84	0.75–0.94	0.002	0.85	0.79–0.92	< 0.001
Current smoker (irregular)	1.07	0.94–1.22	0.31	0.86	0.76–0.97	0.01	0.93	0.85–1.03	0.16
Current smoker (regular)	0.83	0.73–0.95	0.01	1.01	0.91–1.12	0.81	0.98	0.90–1.06	0.55
**Alcohol drinking**									
Never									
Ex-drinker	1.11	0.93–1.33	0.23	0.99	0.84–1.16	0.87	1.16	1.05–1.29	0.01
Current drinker (irregular)	0.84	0.74–0.96	0.01	1.15	1.04–1.28	0.01	1.15	1.07–1.24	< 0.001
Current drinker (regular)	1.16	0.95–1.41	0.15	1.25	1.06–1.47	0.01	1.23	1.09–1.40	0.001
**Exercise**									
No									
Irregular exercise	1.19	1.01–1.41	0.04	1.03	0.89–1.19	0.70	0.88	0.78–0.98	0.02
Regular exercise	0.83	0.70–0.98	0.03	0.87	0.76–1.00	0.06	0.75	0.68–0.84	< 0.001

*HR* Hazard ratio, *95% CI* 95% confidence interval.

### Sensitivity analysis

In the sensitivity analysis, the incidence rate of metabolic syndrome, according to IDF 2005, was 4.3 per 100 person-years (95% CI 4.3–4.4). The incidence rates among males and females were 4.4 per 100 person-years (95% CI 4.3–4.4) and 4.1 per 100 person-years (95% CI 3.9–4.3) (supplement Table 1). We found a consistent association between demographic and behavioral factors. RTA personnel over 35 years had a higher incidence of metabolic syndrome than younger participants with a dose–response relationship. Regarding behavioral factors, the RTA personnel who reported regular exercise had a lower incidence rate of metabolic syndrome than sedentary participants. In contrast, alcohol intake was not associated with the incidence rate of metabolic syndrome according to IDF 2005 (supplement Table 2).

## Discussion

To our knowledge, the present study is the largest recent epidemiologic study of the incidence of metabolic syndrome among RTA personnel in Thailand. These data provide essential evidence on behavioral risk factors for metabolic syndrome in this population.

Several studies have reported data on the prevalence of metabolic syndrome in diverse populations in different regions of the world. However, the results of these studies are difficult to compare because of variation in the criteria used to define metabolic syndrome, with some relying on the NCEP ATP III, the IDF, and the World Health Organization (WHO)^2^ criteria.

We found that the overall incidence rate of metabolic syndrome among RTA personnel was 3.7 per 100 person-years. This rate is relatively high compared with results from a nine-year follow-up study in a rural community in Thailand, reporting an incidence rate of metabolic syndrome of 3.5 per 100 person-years^25^. Furthermore, this incidence rate was higher than that reported in a Japanese^7^ and a Taiwanese^8^ cohort indicating a rate of 2.1 and 2.3 per 100 person-years, repectively. This finding may be explained by the institutional structures of the military that convey detrimental behaviors and weaken personnel’s efforts to embrace healthier habits^14^; for example, in the present study a higher prevalence was found of regular alcohol consumption compared with the study participants in a Japanese^7^ and a Taiwanese^8^ cohort.

When considering the association between sex and the incidence of metabolic syndrome, our finding of a higher rate among males than females was consistent with results from a Japanese cohort^7^, but other studies, including a cohort of US military personnel^26^, and cohorts from Korea^27^ and Thailand^25^ have reported higher rates among females than males. Beliefs about masculinity embedded in the culture of military personnel may establish the behavioral patterns of men in styles affecting their health awareness^28^. Compared with females, on average, males have higher risk for cardiovascular diseases^29, 30^ and premature death^31^. The majority of the RTA population is male; therefore, targeting behaviors, that may mitigate the disparity in metabolic syndrome incidence between males and females has the potential to lower the incidence of sequelae of metabolic syndrome, including NCDs and ASCVD in particular.

In the present study, we intended to determine associations between the incidence rate of metabolic syndrome and behavioral factors at baseline. According to NCEP ATP III (2005), metabolic syndrome consisted of laboratory testing, history of pharmacologic treatment and anthropometric measurements. The RTA personnel, after participating in the annual physical health examination, received their health examination results and may have been advised by healthcare workers to modify their lifestyle. If the RTA personnel improved their health behaviors, it may have positively affected their laboratory testing results and anthropometric measurements during the follow-up period. Therefore, the incidence rates of metabolic syndrome in this population may have been underestimated.

We found that participants older than 35 years exhibited a higher incidence of metabolic syndrome than younger participants with a dose–response relationship in both sexes. Although our findings were consistent with a report from Japan reporting a higher incidence of metabolic syndrome among older than younger adults, a few studies did not find an increase of metabolic syndrome with increasing age^25, 27^, suggesting that the association with age was not inevitable. Several mechanisms can explain a higher incidence of metabolic syndrome with increasing age. For example, central adiposity, accumulation of fatty infiltration of the liver and sedentary lifestyle have a higher prevalence among older than younger individuals^32^. Furthermore, an age-related increase in oxidative stress plays a significant role in contributing vascular alterations by triggering the biochemical processes concomitant with metabolic syndrome^33–35^. When stratified by sex, the incidence rate of metabolic syndrome among participants aged ≥ 45 years was higher than those aged < 35 years with adjusted HR 6.34 (95%CI 6.01 to 6.70) for males, and 9.59 (95% CI 7.55 to 12.19) for females. Alterations in circulating female sex hormone levels, such as lower estrogen levels, were associated with distinct changes in adipose distribution patterns, reverting to visceral accumulation and raising the likelihood of increases in WC^19, 36^. Therefore, our study suggested that preventing the development of metabolic syndrome with increasing age is an essential target for the primary prevention of NCDs and ASCVD later in life.

Excessive alcohol consumption is a known behavioral risk factor for the incidence of metabolic syndrome and NCDs^37, 38^. This study found that participants reporting alcohol consumption (regular or irregular) were more likely to develop metabolic syndrome than abstainers. A recent report from a contemporary Japanese cohort reported that compared with abstainers, consumption of more than 60 g of alcohol daily was associated with a higher rate of metabolic syndrome. In contrast, less than 20 g of alcohol daily was associated with a lower risk^16^. In 2019, The 6th National Health Examination Survey (NHES VI) in Thailand reported that the prevalence of alcohol consumption among Thai adults was 44.6, 59.0 and 31.0% in total, and among males and females, respectively^16^. Compared the NHES VI, our findings indicated that the prevalence of current alcohol consumption was higher among RTA personnel (70.7%) than that of the general Thai population^16^. Therefore, alcohol consumption may be an important contributor to the incidence of metabolic syndrome, especially among male RTA personnel. According to tradition among males RTA personnel, reduced harmful use of alcohol may constitute a priority^39^. Therefore, additional pattern and intensity of alcohol consumption should be assessed in the annual physical health examination section; then motivational interventions such as a Brief Negotiated Interview should be offered to support consumers to modify their risky behaviors^40^.

We found that compared with lifelong nonsmokers, former smokers had a lower incidence of metabolic syndrome. This finding may result from the lifestyle modification among former smokers who may have relatively higher health awareness than lifelong nonsmokers. For example, a related study in China reported that former smokers had significantly more knowledge of all health effects than those who had never smoked^41^. Stratified by age, younger RTA personnel, aged < 35 years, compared with lifelong non-smokers, current smokers exhibited a lower incidence of metabolic syndrome. This observation was likely due to the well-documented negative relationship between smoking and obesity^42, 43^ and the finding that current smokers have less subcutaneous and visceral adipose tissue than those who never have smoked^38, 44^. However, not all studies have found a negative association between smoking and metabolic syndrome. For example, a report from the CARDIA study in the US did not find a significant association between smoking and risk of metabolic syndrome (RR 1.16; 95% CI 0.96 to 1.40)^45^. Furthermore, a meta-analysis of prospective studies found an overall significantly positive association between active smoking and the incidence of metabolic syndrome^46^.

In terms of regular exercise, RTA personnel may have more physical activity than the general civilian population^16^. However, RTA personnel serve in various departments with different characteristics of duty, for instance, military training units, healthcare workers in military hospitals and office workers in headquarters. Hence, the level of physical activity varied across our study population. We found that regular exercise was associated with a lower incidence of metabolic syndrome among RTA personnel. Regular exercise was negatively associated with obesity, a component of metabolic syndrome^47^. Therefore, our study suggested that regular exercise should be encouraged to lower the incidence of metabolic syndrome and ultimately NCDs, including ASCVD^48, 49^. Because vigorous physical activity can rarely trigger acute cardiovascular events or heat-related illness^50–52^, physical activity and structured exercise should be performed based on relevant guidelines^53^.

This study encountered several limitations. First, the present study was conducted among RTA personnel comprising a greater proportion of male participants than that of the general population. However, the results reported the real-world situation in the RTA population.

Because this constituted a retrospective cohort study using previously collected data, some variables were collected very broadly. For example, we did not have detailed data on how many days per week participants consumed alcohol or the number of alcoholic beverages consumed daily. Likewise, we did not have details of the smoking history, such as the current number of cigarettes smoked daily or pack-years of past exposure. We also did not have detailed data on the frequency, type or intensity of physical activity. Because we used collected data, unmeasured confounders such as family history, socioeconomic status, total calorie intake and nutritional status were excluded in the analysis. Due to the nature of an observational study, the information on some variables was unobtainable, including smoking status (5.0%), alcohol consumption (4.5%) and exercise (5.7%). Nonetheless, the available data provided valuable evidence regarding the associations between these health behaviors and the incidence of metabolic syndrome. The information on laboratory testing and anthropometric measurements came from several RTA hospitals nationwide providing the measurements; however, the measurements were performed by trained technicians, and the standard and quality of services of all RTA hospitals were certified by Healthcare Accreditation Institute, Thailand. In the present study, we aimed to determine associations between the incidence rate of metabolic syndrome and behavioral factors at baseline. However, the RTA personnel received their health examination results and may have been advised by healthcare workers to modify their lifestyle; thus, their behaviors may be changed over time. Thus, time-varying covariates may be considered to approach in future research.

Our study also exhibited significant strengths; of the approximately 130,000 RTA personnel, 98,264 (75.6%) participants, without a diagnosis of metabolic syndrome at baseline, were enrolled in the present study, representing a large sample of RTA personnel. Thus, our findings provide valuable insights into the demographics and behavioral and clinical risk factors, for the incidence of metabolic syndrome in this population. These data may contribute to strategies for the primary prevention of NCDs, ASCVD and premature death in Thai populations.

## Conclusion

Our data demonstrated that metabolic syndrome is a common health issue, especially among males and RTA personnel over age 35. Alcohol consumption and sedentary behavior appear to play an important role in the incidence of metabolic syndrome in this population and are potential targets for interventions to enhance primary prevention of the sequelae of metabolic syndrome, including NCDs and ASCVD.

## Supplementary Information

Supplementary Information.

## Data Availability

Data cannot be shared publicly because the data set contains identifying information; additionally, the data belongs to the Royal Thai Army Medical Department. Thus, ethics and confidentiality restrictions exist on the distribution of the data set. Data are available from the Royal Thai Army Medical Department, Bangkok, Thailand, for researchers who meet the criteria to access confidential data.

## Abbreviations

NCDs: Noncommunicable diseases
ASCVD: Atherosclerotic cardiovascular diseases
RTA: The Royal Thai Army
H.R.: Hazard ratio
CI: Confidence interval
SD: Standard deviation
