# Supplementary material for: Incidence and risk factors of metabolic syndrome among Royal Thai Army personnel
Source: Sci Rep. 2022 Sep 20;12:15692. doi: 10.1038/s41598-022-19024-8 (PMC9489720; doi:10.1038/s41598-022-19024-8)
Supplement: Supplementary file 1 — Supplementary Information. [file 41598_2022_19024_MOESM1_ESM.pdf]

Supplement Table 1. The incidence of metabolic syndrome (IDF 2005) among RTA personnel by demographic and behavioral factors

	Person-years of observation	No. of metabolic syndrome	Incidence rate / 100 person-years	95% CI	*p*-value
**Total**	281901	12240	4.34	4.27-4.42	
**Sex**					0.06
Female	24862	1022	4.11	3.87-4.37	
Male	257039	11218	4.36	4.28-4.45	
**Age (years)**					<0.001
mean±SD					
<35	157038	2350	1.50	1.44-1.56	
35-44	53419	3633	6.80	6.58-7.03	
≥45	71444	6257	8.76	8.54-8.98	
**Regions**					<0.001
Bangkok	44312	2131	4.81	4.61-5.02	
Central	79522	3520	4.43	4.28-4.58	
Northeast	58548	2834	4.84	4.67-5.02	
North	62566	2262	3.62	3.47-3.77	
South	36953	1493	4.04	3.84-4.25	
**Health Scheme**					0.03
Civil Servant Medical Benefits	277690	12077	4.35	4.27-4.43	
Social Security	2892	123	4.25	3.56-5.08	
Universal Coverage	1319	40	3.03	2.22-4.13	
**Smoking status**					<0.001
Never	139334	6403	4.60	4.48-4.71	
Ex-smoker	50309	2306	4.58	4.40-4.77	
Current smoker (irregular)	38159	1499	3.93	3.73-4.13	
Current smoker (regular)	51370	1938	3.77	3.61-3.94	
**Alcohol drinking**					<0.001
Never	52148	2488	4.77	4.59-4.96	
Ex-drinker	26680	1239	4.64	4.39-4.91	
Current drinker (irregular)	179184	7540	4.21	4.11-4.30	
Current drinker (regular)	22610	926	4.10	3.84-4.37	
**Exercise**					<0.001
No	18996	907	4.77	4.47-5.10	
Irregular exercise	83958	4246	5.06	4.91-5.21	
Regular exercise	175467	6964	3.97	3.88-4.06	

95% CI: 95% confidence interval

Supplement Table 2. Univariable and multivariable analysis of the association between demographic, behavioral, and clinical factors and the incidence of metabolic syndrome (IDF 2005) among RTA personnel

Factors	Unadjusted HR	95% CI	*p*-value	Adjusted HR	95% CI	*p*-value
**Sex**						
Female						
Male	1.06	1.00-1.13	0.07	1.25	1.16-1.34	<0.001
**Age (years)**						
<35						
35-44	4.55	4.32-4.79	<0.001	4.61	4.38-4.86	<0.001
≥45	5.86	5.59-6.14	<0.001	5.95	5.67-6.25	<0.001
**Regions**						
Bangkok						
Central	0.92	0.87-0.97	0.003	1.12	1.06-1.19	<0.001
Northeast	1.01	0.96-1.07	0.605	1.22	1.15-1.29	<0.001
North	0.75	0.71-0.80	<0.001	0.91	0.86-0.97	0.004
South	0.84	0.79-0.90	<0.001	1.28	1.19-1.37	<0.001
**Health Scheme**						
Civil Servant Medical Benefits						
Social Security	0.97	0.81-1.16	0.72	1.10	0.92-1.32	0.30
Universal Coverage	0.67	0.49-0.91	0.01	0.77	0.56-1.06	0.10
**Smoking status**						
Never						
Ex-smoker	1.00	0.95-1.04	0.87	0.99	0.94-1.04	0.72
Current smoker (irregular)	0.85	0.81-0.90	<0.001	1.02	0.96-1.08	0.59
Current smoker (regular)	0.82	0.78-0.86	<0.001	0.92	0.87-0.97	0.004
**Alcohol drinking**						
Never						
Ex-drinker	0.97	0.91-1.04	0.41	0.97	0.9-1.05	0.46
Current drinker (irregular)	0.88	0.84-0.92	<0.001	1.02	0.97-1.07	0.42
Current drinker (regular)	0.85	0.79-0.92	<0.001	0.95	0.88-1.03	0.25
**Exercise**						
No						
Irregular exercise	1.06	0.99-1.14	0.12	1.00	0.93-1.08	0.97
Regular exercise	0.83	0.77-0.89	<0.001	0.78	0.73-0.84	<0.001

HR: hazard ratio: CI: 95% confidence interval
